# A continuing professional development imperative? Examining trends and characteristics of health professions education doctoral programs

**DOI:** 10.1186/s12909-022-03937-z

**Published:** 2022-12-09

**Authors:** Violet Kulo, Christina Cestone

**Affiliations:** Health Professions Education, Graduate School, University of Maryland Baltimore, MD Baltimore, USA

**Keywords:** Health professions education, Continuing medical education, Continuing professional education, Continuing professional development, Faculty development

## Abstract

**Background:**

Despite the long-standing faculty development initiatives for improving teaching skills in the health professions, there is still a growing need for educators who are formally trained in educational theory and practice as health professions schools experience dramatic demand and growth. Graduate programs in health professions education (HPE) provide an avenue for health professions’ faculty continuing professional development to enhance their knowledge and skills for teaching and curriculum leadership roles. There has been a proliferation of certificate, master’s, and doctoral programs in HPE over the last two decades to respond to the growing need for well-prepared faculty educators and program leadership. The purpose of this study was to identify and describe current HPE doctoral programs in United States (U.S.) and Canada.

**Methods:**

The study first examined doctoral programs in HPE identified in earlier studies. Next, we searched the literature and the web to identify new doctoral programs in the U.S. and Canada that had been established between 2014, when the prior study was conducted, and 2022. We then collated and described the characteristics of these programs, highlighting their similarities and differences.

**Results:**

We identified a total of 20 doctoral programs, 17 in the U.S. and 3 in Canada. Of these, 12 programs in the U.S. and 1 program in Canada were established in the last 8 years. There are many similarities and some notable differences across programs with respect to degree title, admission requirements, duration, delivery format, curriculum, and graduation requirements. Most programs are delivered in a hybrid format and the average time for completion is 4 years.

**Conclusions:**

The workforce shortage facing health professional schools presents an opportunity, or perhaps imperative, for continuing professional development in HPE through certificate, master’s, or doctoral programs. With the current exponential growth of new doctoral programs, there is a need to standardize the title, degree requirements, and further develop core competencies that guide the knowledge and skills HPE graduates are expected to have upon graduation.

## Background

Health professions will face dramatic workforce and faculty shortages nationally due to several converging factors: an aging workforce, a growth in care needs of an aging population, and expanding healthcare coverage [1]. For example, medicine will face an anticipated shortage between 54,100 and 139,000 physicians by 2033 [1, 2]. The American Association of Colleges of Nursing cites that on average there will be “175,900 openings for RNs each year through 2029 when nurse retirements and workforce exits are factored into the number of nurses needed in the U.S.” (p. 1) [3]. Further, the Bureau of Labor Statistics anticipates the need for nurse anesthetists, nurse midwives, and nurse practitioners to grow by 40%, registered nurses by 6%, and the need for physician assistants is projected to grow by 28% through 2031 [4–6].

To combat looming attrition due to workforce aging, admissions and enrollment increases are evident across fields, from medicine, nursing, to physician assistants [7–9]. This expansion in the training pipeline exacerbates the pressing problem of faculty shortages that many health professions are already experiencing. For instance, 80,407 qualified applicants were turned away from nursing programs in 2019 mainly due to faculty shortages [10]. In a 2019 program survey, 88% of physician assistant programs reported lack of qualified faculty as a barrier to recruiting new faculty, and 90% of programs reported that candidates lack the required teaching experience [11].

Indeed, a majority of faculty in the health professions have little to no formal training in educational theory and practice, despite more than three decades of calls to develop and reward medical education teachers for their educational work [12]. This gap has traditionally been addressed by faculty development (FD) initiatives aimed at improving teaching skills. Bligh asserted that “successful faculty development results in improved teaching performance and leads to better learning outcomes for students or doctors” (p. 120) [13]. There have been long-standing FD initiatives in the health professions, with longitudinal programs cited as having sustained positive effects on intrapersonal, interpersonal, teaching, and career development [14].

In systematic reviews of FD literature, researchers reported numerous studies on initiatives designed to improve teaching effectiveness and leadership abilities in medical education [15–19]. They noted that participants’ knowledge of educational principles and specific instructional strategies increased, and gains in teaching skills as well as changes in instructional practices were reported [15, 16]. Similarly, for FD interventions designed to improve leadership abilities, participants reported changes in leadership behavior in addition to gains in leadership knowledge and skills [18]. Ward and Stanulis proposed a model for FD using targeted coaching and assisted performance to help medical educators learn to teach effectively [20].

In nursing education, research on FD initiatives found an increase in participants’ knowledge and skills as well as improved preparation for clinical teaching [21–23]. With regard to FD initiatives in dental education, participants reported increased knowledge of instructional innovation, improved instructional practice, and implementation of new instructional methods [24]. Tricio and colleagues found that dental faculty used more student-centered strategies and reported improved quality of student learning, whereas students reported improved teaching skills [25]. In a program to teach pharmacy faculty the concept of a flipped classroom, participants reported increased understanding of a flipped classroom and stated that they would consider flipping one of their classes [26].

Despite the faculty development initiatives for improving teaching practices, challenges remain in improving teaching skills and educational knowledge among health professions educators who may benefit. In response to the challenge, there is a proliferation of graduate programs in health professions education (HPE) to address the need for educators who are formally trained in teaching and learning [27, 28]. Additionally, researchers contended that HPE programs can strengthen the preparation of continuing professional development (CPD) educators and enhance the delivery of CPD by providing the knowledge, skills, and competencies needed to teach and lead effectively [29, 30]. Further, with the ongoing debate on whether physician assistant (PA) education should transition to an entry-level doctoral degree [31, 32], HPE programs can provide an avenue for preparing doctorally-trained PA faculty.

Over the last two decades, the number of graduate programs in HPE has grown tremendously including certificate, master’s, and doctoral programs. The master’s degree has been the most chosen credential [33] and has the potential to facilitate the development of a critical mass of educators and thus, meet the need for faculty supply. In addition to preparing skilled faculty educators, doctoral programs cultivate independent researchers to advance theory and improve practice through conducting rigorous educational research and scholarship. A study conducted in 1998 identified a total of 15 HPE programs worldwide [34] and subsequent studies have been conducted with the most recent studies in 2012 and 2014 identifying 121 master’s programs and 24 doctoral programs, respectively [33, 35–38]. A list of existing master’s and PhD programs in HPE worldwide including their delivery format and links to program websites was previously maintained by the Foundation for Advancement of International Medical Education and Research (FAIMER) [39]. However, FAIMER did not provide a detailed description of the characteristics of these programs. New doctoral programs have launched since the 2014 study but a comprehensive description of these programs to guide prospective HPE students and CPD educators is lacking. The purpose of this study was to 1) examine all current HPE doctoral programs in the U.S. and Canada, 2) identify any new doctoral programs that have been established since the prior study in 2014, and 3) describe the characteristics of these programs, highlighting their similarities and differences.

## Methods

Our analysis began with examining HPE doctoral programs identified in earlier work [37]. We then reviewed the FAIMER website to verify if any new programs had been added to the site since the article was published. In our initial review in the Summer of 2020, the FAIMER website had not yet been updated with newer programs but was still available. Therefore, we conducted an online search using the following search terms: “PhD in HPE,” “EdD in HPE,” “doctoral/doctorate programs in HPE,” “doctoral/doctorate degree in HPE,” and “doctoral/doctorate and HPE.” Using the same search terms, we conducted a literature review in PubMed, ERIC, and Google Scholar to find any additional articles describing HPE doctoral programs.

Our inclusion criterion was HPE doctoral programs in the U.S. and Canada because we wanted to understand the similarities and differences of North American programs. We excluded programs that did not focus on education in the health professions. We collected the characteristics of the programs from each program’s website. In the cases where information was no longer available online for programs that were identified in the previous study, we contacted administrators at these institutions to verify whether the program was still being offered. We organized program information in a table consisting of the following 10 fields: name and location of institution, degree title, admission requirements, published program duration, credit hours, graduation requirements, delivery format, program cost, academic location, and website link.

## Results

We identified 20 doctoral programs, 17 in the U.S. and 3 in Canada (Table 1). Since 2014, 12 new programs have been established in the U.S. and 1 new program in Canada. We also found that the PhD program at North Carolina State University no longer exists. Additionally, while two EdD programs identified in the previous study still exist, they no longer offer a concentration in HPE and were therefore excluded from the results (College of Saint Mary and Nova Southeastern University). The characteristics of the 20 programs, as well as their similarities and differences, are described next.

**Table 1:** Characteristics of HPE doctoral programs in the U.S. and Canada

**Institution** **United States**	**Degree title**	**Admission requirements**	**Published program duration**	**Credit hours**	**Graduation requirements**	**Delivery format**	**Program cost**	**Academic location**	**Website**
A.T. Still University – Mesa, AZ	EdD in Health Professions	• Master’s degree (GPA ≥ 2.5/4.0) • Curriculum vitae • Essay • TOEFL scores if applicable	3 years	55	• Complete required courses • Research project	Online	$691 per credit hour	College of Graduate Health Studies	https://www.atsu.edu/doctor-of-education-in-health-professions-degree
Allen College – Waterloo, IA	EdD in Health Professions Education	• Master’s degree or higher (GPA ≥ 3.0/4.0) • Completion of a statistics course • Interview may be required • Proof of English proficiency if applicable • Three recommendations • Writing sample • Statement of purpose	3–4 years	60	• Complete required courses • Education-focused capstone project	Hybrid	$670 per credit hour	School of Health Sciences	https://www.allencollege.edu/doctor-of-education.aspx
Bellarmine University – Louisville, KY	PhD in Health Professions Education	• Master’s degree or professional doctorate (GPA ≥ 3.2/4.0) • Minimum of 1 year in educator role and/or 3 years in a health professions practice role • Two references • Essay addressing professional goals • Curriculum vitae • Professional license • Interview • TOEFL scores if applicable	3 years	48	• Complete required courses • Teaching practicum • Contemporary concepts seminars • Pre-dissertation seminar • Dissertation	Online	$855 per credit hour	College of Health Professions	https://www.bellarmine.edu/health-professions/graduate/phd-in-health-professions-education/
D’Youville University – Buffalo, NY	EdD in Health Professions Education	• Master’s degree (GPA ≥ 3.0/4.0) • Two letters of recommendation • Statement of goals • Curriculum vitae • Writing sample • Interview	3 years	60	• Complete required courses • Practicums • Dissertation	Online	$950 per credit hour	School of Health Professions	http://www.dyc.edu/academics/schools-and-departments/health-professions/programs-and-degrees/health-professions-education-edd.aspx
Eastern Virginia Medical School – Norfolk, VA	EdD/PhD in Medical and Health Professions Education	• Master’s degree (GPA ≥ 3.0/4.0) • One year teaching in medical health professions/higher education • Personal essay • Writing sample • Curriculum vitae • Two references • TOEFL scores if applicable	3.5-4 years	48	• Complete required courses • Dissertation	Online	$956 per credit hour	School of Medicine	https://www.evms.edu/education/medical_and_health_professions_education/mhpe_doctoral_program/
Logan University – Chesterfield, MO	DHPE – Doctor of Health Professions Education	• Master’s degree or higher (GPA ≥ 3.0/4.0) • Curriculum vitae • Clinical license if applicable	3–4 years	60	• Complete required courses • 2 preceptorship/ practicum • Applied research project	Online	$650 per credit hour	College of Health Sciences	https://www.logan.edu/academics/doctorate-health-professions/
MGH Institute of Health Professions – Boston, MA	PhD in Health Professions Education	• Master’s degree or higher • Statement of goals • Curriculum vitae • Two letters of recommendation • TOEFL scores if applicable	4 years	66	• Complete required courses • Onsite seminars • Dissertation	Hybrid	$1342 per credit hour	Center for Interprofessional Studies and Innovation	https://www.mghihp.edu/phdhped#node-177726
Rocky, Mountain University – Provo, UT	PhD in Health Sciences with concentration in Healthcare Professions Education	• Master’s or professional practice degree (GPA ≥ 3.4/4.0) • Two letters of recommendation • Statement of goals • Curriculum vitae • B- or better in a research methods or statistics course • ≥ 1 year in clinical practice • Healthcare license	4 years	70	• Complete required courses • Onsite sessions • Dissertation	Online	$944 per credit hour	College of Health Sciences and Lifelong Learning	https://rm.edu/phd/
Seton Hall University – South Orange, NJ	PhD in Health Sciences	• Master’s degree (GPA ≥ 3.0/4.0) • Two letters of recommendation • Statement of goals and research/career interests • Curriculum vitae • Interview may be required • GRE scores if available • TOEFL scores if applicable	4–8 years	57	• Complete required courses • Dissertation	Hybrid	$1354 per credit hour	School of Health and Medical Sciences	https://www.shu.edu/academics/phd-health-sciences.cfm
Simmons College – Boston, MA	PhD in Health Professions Education	• Master’s degree (GPA ≥ 3.0/4.0) • Curriculum vitae • GRE scores • Three letters of recommendation • Writing sample • Personal statement • TOEFL scores if applicable	4 years	48	• Complete required courses • Capstone/Placement/Internship/Practicum • Dissertation	Hybrid	$1280 per credit hour	School of Nursing	https://www.simmons.edu/graduate/academic-programs/graduate-and-certificate-programs/health-professions-education-phd
Uniformed Services University – Bethesda, MD	PhD in Health Professions Education	• Military service or federal employee • Curriculum vitae • Three letters of recommendation • Personal statement • Research statement • GRE scores	4 years	144	• Complete required courses • Portfolio • Practicum • Oral exam • Dissertation	Hybrid	Free for military and federal employees	School of Medicine	https://chpe.usuhs.edu/graduate-programs/phd-in-hpe
University of California San Francisco, CA / Utrecht University Medical Center, Netherlands	PhD in Health Professions Education	• Health professional with preparation in education or master’s degree in social or biomedical sciences • UCSF faculty or staff involved in health professions education • Publications in education research desirable	Maximum 6 years		• Complete required training activities • Monthly cohort virtual meeting • Annual progress interview in San Francisco • Thesis comprising 4–6 published research studies in peer-reviewed international journals • Thesis defense in Netherlands	Hybrid	A total of three tuition payments	School of Medicine	https://meded.ucsf.edu/faculty-educators/faculty-development-all/advanced-education-programs/doctoral-program-health-professions-education
University of Houston, TX	EdD in Professional Leadership for Health Science Education	• Master’s degree (GPA ≥ 2.6/4.0) • GRE scores • Statement of interest • Curriculum vitae • Writing sample • Letters of recommendation • TOEFL scores if applicable	3 years	51	• Complete required courses • Dissertation	Hybrid	Resident: $617 per credit hour Non-resident: $967 per credit hour	College of Education	http://medical.coe.uh.edu/executive-doctorate.htm
University of Illinois Chicago, IL	PhD in Critical Pedagogies and Urban Teacher Education with a specialization in Health Professions Education	• Bachelor’s or master’s degree • Goal statement • Curriculum vitae • Three letters of recommendation • TOEFL scores if applicable	4–6 years	96/64	• Complete required courses • Research project • Written and oral preliminary exams • Dissertation	Hybrid	$838 per credit hour	College of Education	https://education.uic.edu/academics/programs/critical-pedagogies/
University of Maryland Baltimore, MD	PhD in Health Professions Education	• Master’s degree • Statement of proposed research • Curriculum vitae • Three letters of recommendation • Clinical license if applicable • Interview • TOEFL scores if applicable	3–5 years	60	• Complete required courses • Onsite summer impact institute • Dissertation	Online	Resident: $761 per credit hour Non-resident: $972 per credit hour	Graduate School	https://graduate.umaryland.edu/healthprofessionsedPhD/
University of Texas at El Paso, TX	PhD in Interdisciplinary Health Sciences	• Master’s degree • GRE scores • TOEFL scores if applicable • Three letters of recommendation • Statement of goals and research interest • Interview	3 years	60	• Complete required courses • Dissertation	Face-to-face	Resident: $505.05 per credit hour Non-resident: $1020.50 per credit hour	College of Health Sciences	https://www.utep.edu/chs/ihs/
Widener University – Chester, PA	PhD in Health Professions Education	• Master’s degree (GPA ≥ 3.0/4.0) • Statement of interest • Curriculum vitae • One letter of recommendation	3 years	57	• Complete required courses • Teaching practicum • Dissertation	Hybrid	$990	College of Health & Human Services	https://www.widener.edu/academics/graduate-studies/health-professions-education-phd
**Institution** **Canada**	**Degree title**	• **Admission requirements**	**Published program duration**	**Credit hours**	• **Graduation requirements**	**Delivery format**	**Program cost**	**Academic location**	**Website**
University of Calgary, AB	PhD in Community Health Sciences: Medical Education	• Master’s degree (GPA ≥ 3.3/4.0) • Statement of research interest • Letter of interest from a faculty member to be a supervisor • Two letters of recommendation • Completion of core courses and specialization courses or equivalents required at the master's level • Relevant work and/or research experience recommended • TOEFL scores if applicable	4–6 years	9 units minimum	• Complete required courses • Attend weekly Medical Education Journal Series and seminars • Thesis	Face-to-face	Citizens/residents: $3464 per year International: $8081 per year	School of Medicine	https://grad.ucalgary.ca/future-students/explore-programs/community-health-sciences-phd
University of Ottawa, ON	Ph.D. in Education (Health Professions Education)	• Master’s degree with thesis in science, health sciences or related field • Minimum average of B+ • Letter of intent • Curriculum vitae • Two letters of recommendation • TOEFL scores if applicable	5–6 years	18 units minimum	• Complete required courses • Thesis	Face-to-face	Citizens/residents: $8091 per year International: $8811 per year	Faculty of Education	https://catalogue.uottawa.ca/en/graduate/doctorate-philosophy-education-concentration-health-professions-education/
Western University – London, ON	PhD in Health Professions Education	• Master’s degree • Statement of research interest • A cover letter • Curriculum vitae • Two letters of recommendation • TOEFL scores if applicable	4 years	1.5 credits for courses only	• Complete required courses • Attend and participate in seminars • Thesis	Face-to-face	Citizens/residents: $8499 per year International: $9219 per year	Faculty of Health Sciences	https://www.uwo.ca/fhs/programs/hrs/fields/health_pro_edu.html

### Location of institution and degree title

**Fig. 1:**
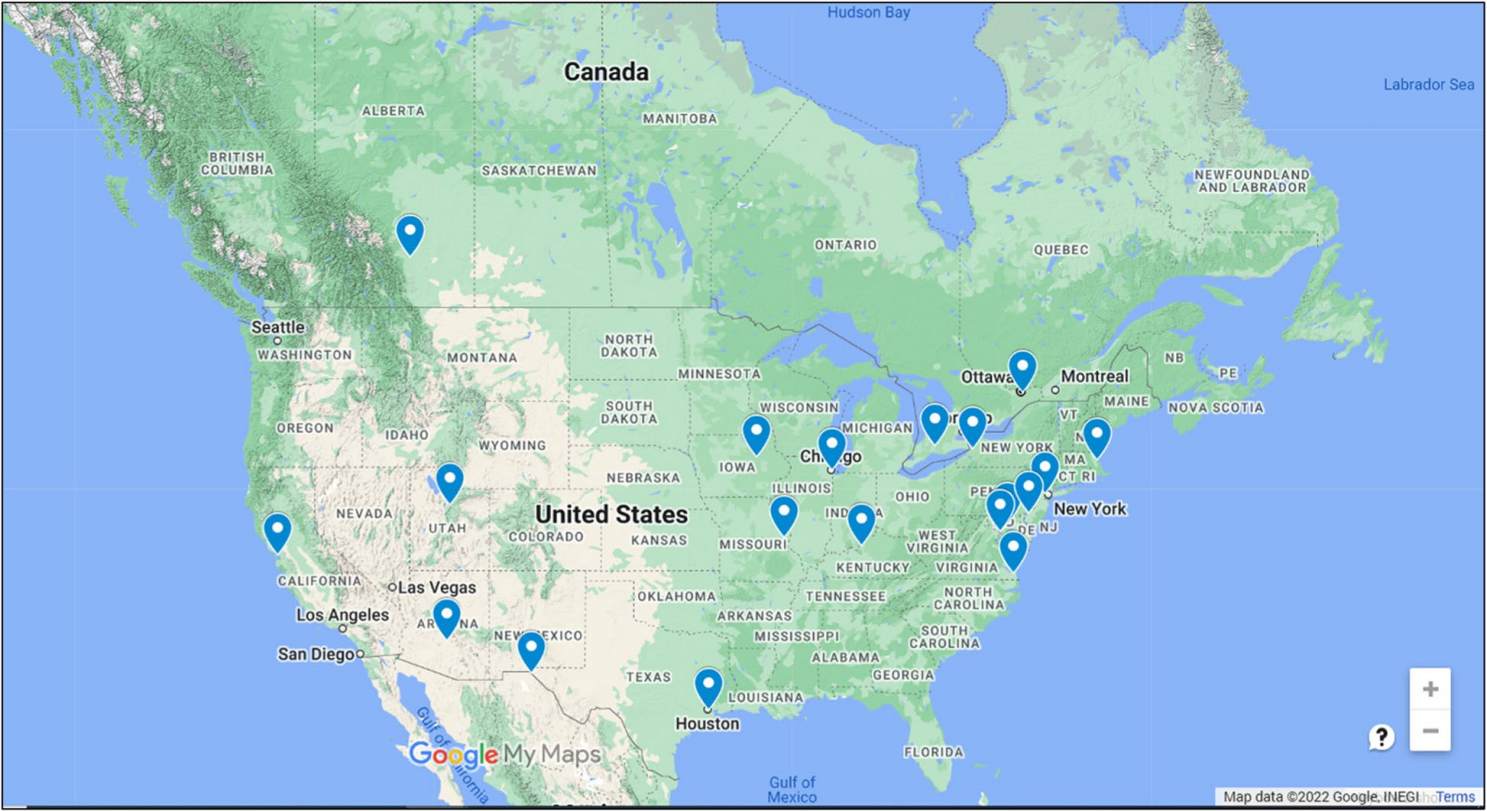
Geographic locations of HPE doctoral programs in the U.S. and Canada

Using the geographic classification system of the U.S. Census Bureau, 6 of the 17 U.S. institutions are in the South, 5 in the Northeast, 3 in the Midwest, and 3 in the West [40]. Using the census divisions of Canada, 2 programs are located in Ontario and 1 in Alberta [41]. Figure 1 maps the geographic locations of all 20 programs [42].

Eight of the 20 programs are doctor of philosophy degrees (PhD) in HPE, three programs offer an educational doctorate (EdD) in HPE, two programs offer a PhD in Health Sciences, and one offers a Doctor of Health Professions Education (DHPE). There were other notable variations in offerings and title of the degree. These differences include: Eastern Virginia Medical School (EVMS) offers both an EdD and PhD in Medical and Health Professions Education, University of Houston (UH) offers an EdD in Professional Leadership for Health Science Education, University of Illinois Chicago (UIC) offers a PhD in Education with a concentration in Critical Pedagogies and Urban Teacher Education and a specialization in Health Professions Education. The University of Texas at El Paso (UTEP) offers a PhD in Interdisciplinary Health Sciences, University of Ottawa offers a PhD in Education with a concentration in HPE, whereas the University of Calgary offers a PhD in Community Health Sciences with a specialization in Medical Education.

### Admission requirements

The criteria for admissions also varied across institutions. Nineteen programs (95%) require a master’s degree or higher for admission. UIC also admits students with a bachelor’s degree, however these students must complete additional coursework. Ten programs require a grade point average (GPA) of 3.0 and above, one program requires a minimum GPA of 2.6 and another requires a minimum GPA of 2.5. Seventeen programs require a statement of purpose, 16 require a curriculum vitae, 15 require letters of recommendation, and 5 require a writing sample. Interviews are required for four programs and may be required for two programs. Four programs require the Graduate Record Examination (GRE) scores, one program recommends GRE scores if available, and 17 programs require the Test of English as a Foreign Language (TOEFL) scores for international applicants.

### Program duration and curricula foci

The duration of these programs ranges from 3 to 8 years depending on whether students are enrolled full-time or part-time. The required credit hours across the U.S. programs averages 58 credit hours, with a range of 48–70 credit hours. One outlier that was not factored into our average requires 144 credit hours. The Uniformed Services University (USU) courses are based on quarter credit hours instead of semester hours, thus, 144 credit hours are equivalent to 96 semester hours. The wide difference in the credit hours is because the USU program requires more courses, as well as more credit hour requirements, for a practicum (20) and dissertation (30). The required credits for the three programs in Canada range from 1.5 credits (courses only) to a minimum of 18 credits. Western University has three mandatory half-credit courses and the rest of the courses are seminars.

Within curricula, most programs include core content areas for HPE programs as previously reported [33]. These include teaching and learning, curriculum development, program evaluation and learner assessment, educational research methods, and leadership and management, with some variation in courses. Some notable courses that are offered in more than one program include, finance and fiscal management/budgeting; cultural competence and multicultural education; diversity in education/healthcare education and the workforce; and professional, ethical, and legal issues in HPE.

Graduation requirements typically include completing required courses, which range from 6–24 courses across the programs, completing a research project, or dissertation/thesis. Three online programs have a residency requirement and onsite seminars. In six programs, students complete a teaching or leadership practicum/internship/preceptorship. The USU program requires students to assemble a comprehensive portfolio that demonstrates they have achieved each HPE program competency.

### Delivery format and cost

The delivery format of the programs varies. Nine programs offer the degree in a hybrid format, combining online and face-to-face instruction. Seven programs are online, and four programs offer only face-to-face instruction. The average cost of U.S. programs is $894 (USD) per credit hour and is $6,685 (CAD) per year in Canada. The current cost of U.S. programs ranges from $505 to $1,354 per credit hour, with the face-to-face program having the least expensive in-state tuition. Of the three programs in Canada, the least expensive one is about $3,464 (CAD) per year and the costliest one is approximately $8,499 per year for citizens/residents and $8,081 - $9,219 per year for international students.

### Academic home

Five programs are hosted in the college/school of medicine, four programs in the college/school of health sciences, two programs each are in the college of education and the college/school of health professions. The remaining seven programs vary or include more than one college or school. One program each is housed in a graduate school, center for interprofessional studies and innovation, school of nursing, college of graduate health studies, school of health and medical sciences, and college of health and human services. The UIC program is co-hosted by the colleges of education and medicine.

## Discussion

This study examined trends in the number and characteristics of doctoral programs in HPE, comparing their similarities and differences. We found that there were 10 programs in the U.S. and Canada in 2014 and we identified 13 new programs that had been established since that time – more than 100% increase in a span of 8 years. Our search revealed, however, that three of the initial eight programs in the U.S. no longer offer a doctoral degree in HPE. It is unclear why two of the programs discontinued the HPE concentration and one program stopped offering the degree completely.

Most programs in the U.S. are in the Southern region with the Western region having the fewest programs. It is worth noting that using the same geographic classification system, we classified U.S. medical schools [43] and the results were strikingly similar; the South region has the highest number of medical schools, and the West region has the fewest. The congruence between the number of doctoral programs in HPE and medical schools by region may reflect the fact that more institutions are responding to the increasing demand for health professions educators who have formal training in education, as reflected in the Southern region. While most of the information about the programs was available on the program websites, some information was not clear or was incomplete. For example, for admission requirements, some programs did not provide grading criteria or information about the minimum required scores for GRE or TOEFL. This may suggest that these were not important criteria for admission, or they were merely omitted from the website.

There are many similarities across programs with respect to the admission requirements, duration, delivery format, curriculum, and graduation requirements. Almost half of the programs are delivered in a hybrid format, with program duration ranging from 3 to 8 years. Most programs require completion of core courses, elective courses, and either a research project or dissertation/thesis. Of note, there were four unique courses required in some programs including, cultural competence and multicultural education; diversity in healthcare education; finance and fiscal management/budgeting; and professional, ethical, and legal issues in HPE.

While two programs have stand-alone courses in cultural competence and diversity, it is possible that other programs cover these content areas longitudinally across their courses. Since HPE doctoral programs are not overseen by an independent accreditor, there is no “gold standard” for the learning outcomes and the curriculum structure. There is a lot of institutional leeway in the curriculum elements, admissions criteria, program length, and graduation requirements. The variation in curricula begs the question of whether there should be formal standardized competencies for HPE graduates. With the current growth of recent programs, there is a growing need for developing a core body of knowledge or competencies to provide guidance on the knowledge and skills all HPE graduates should possess. Researchers recently proposed seven domains of competence for the HPE doctoral curriculum, including professional expert, research and scholarship, teaching, interdisciplinary collaboration, leadership and management, professionalism, and personal and professional development [44]. Another study suggested 24 entrustable professional activities (EPAs) for HPE graduates categorized into three broad areas: research and scholarship, educational development, and educational management [45]. External validity of the 24 EPAs resulted in 17 EPAs across two domains, namely, research and scholarship and educational development [46]. These competencies and EPAs need to be pilot tested with HPE doctoral students in different programs to validate them.

## Recommendations

As described earlier, there is a wide variation in the current titles given to HPE programs. For example, PhD in Medical Education, PhD in Health Sciences, EdD in Professional Leadership for Health Science Education, and PhD in Community Health Sciences: Medication Education. Some of the titles may not be noticeably clear to prospective applicants regarding the focus of the degree. This was sometimes unclear to the authors without carefully reading the program and course titles or descriptions. We recommend that the naming convention of the degree be standardized to include “Health Professions Education” in the title.

In recent articles, several authors have contended that HPE should be considered as its own field because 1) it meets Boyer’s four criteria of a mature scholarly discipline namely discovery, integration, application, and teaching [47], and 2) HPE programs have the six characteristics of a distinct academic discipline as described in Krishnan's framework [48]. Therefore, standardizing the degree title to include “Health Professions Education” will bolster the discipline as its own field.

We also recommend that the interprofessional team of faculty who teach in HPE programs includes faculty with expertise in educational theory and practice, in addition to faculty from the health professions fields. Faculty with a background in education can contribute to teaching courses in which they were trained such as, learning theories, curriculum development and instructional design, instructional strategies, and assessment. These are the anchor courses present in most curricula we examined in HPE programs and warrant being taught by subject matter experts.

## Limitations

This study has several limitations. First, it only looked at doctoral programs in U.S. and Canada, which does not provide the complete landscape of doctoral programs worldwide. A second limitation is that the information presented was mostly extracted from program websites and it is possible that this information may not be current for that program. Information about some of the programs was not easy to find on the program websites, which for advancing HPE education, may discourage or limit the number of potential HPE applicants. As much as this article has attempted to provide accurate details about current programs, new programs already in development will emerge in the next few years making this information obsolete. In fact, during this study, we realized that one program had been discontinued after identifying it in our initial search, and then we found four new programs in subsequent searches.

## Conclusions

As health professions experience dramatic demand and growth, the pressing problem of faculty shortages continues to persist. This workforce problem presents an opportunity, or perhaps imperative, for continuing professional development in HPE through certificates, master’s, or doctoral programs. In addition, doctoral programs prepare independent researchers to advance educational theory and practice through research and scholarship. While the current HPE doctoral programs in the U.S. and Canada have many similarities, there are some notable differences with respect to the admission requirements, duration, delivery format, curriculum, cost, and graduation requirements. There is a need for standardizing the degree requirements, title, and developing core competencies to provide guidance on the knowledge and skills HPE graduates are expected to have upon degree completion. Future studies should examine the outcomes from these programs and the impact graduates are making in the HPE field.

## Data Availability

The datasets used and/or analyzed during the current study are available from the corresponding author on reasonable request.

## Abbreviations

CPD: Continuing Professional Development
DHPE: Doctor of Health Professions Education
EdD: Doctor of Education
EPAs: Entrustable Professional Activities
ERIC: Education Resources Information Center
EVMS: Eastern Virginia Medical School
FD: Faculty Development
FAIMER: Foundation for Advancement of International Medical Education and Research
GPA: Grade Point Average
HPE: Health Professions Education
PA: Physician Assistant
PhD: Doctor of Philosophy
RN: Registered Nurse
TOEFL: Test of English as a Foreign Language
UH: University of Houston
UIC: University of Illinois Chicago
USU: Uniformed Services University
UTEP: University of Texas at El Paso
